# Supplementary material for: Molecular tracking devices quantify antigen distribution and archiving in the murine lymph node
Source: eLife. 2021 Apr 12;10:e62781. doi: 10.7554/eLife.62781 (PMC8116055; doi:10.7554/eLife.62781)
Supplement: Transparent reporting form [file elife-62781-transrepform.pdf]

eLIFE

1st Floor
24 Hills Road
Cambridge CB2 1JP, UK

P 01223 855340
W elifesciences.org
T @elife

eLife's transparent reporting form

We encourage authors to provide detailed information *within their submission* to facilitate the interpretation and replication of experiments. Authors can upload supporting documentation to indicate the use of appropriate reporting guidelines for health-related research (see EQUATOR Network), life science research (see the BioSharing Information Resource), or the ARRIVE guidelines for reporting work involving animal research. Where applicable, authors should refer to any relevant reporting standards documents in this form.

If you have any questions, please consult our Journal Policies and/or contact us: editorial@elifesciences.org.

Sample-size estimation

- You should state whether an appropriate sample size was computed when the study was being designed
- You should state the statistical method of sample size computation and any required assumptions
- If no explicit power analysis was used, you should describe how you decided what sample (replicate) size (number) to use

Please outline where this information can be found within the submission (e.g., sections or figure legends), or explain why this information doesn't apply to your submission:

Statistical analysis was done using either a non-parametric 2 tailed Mann-Whitney t-test or multiple t-tests with a two stage step up up method of Benjamini, Krieger and Yekutieli without assuming consistent standard deviations. Each in vivo analysis was performed with 3-6 mice per group as determined by a power calculation using the assumption (based on prior data) that there will be at least a two fold change with a standard deviation of less than 0.5. To calculate numbers we performed a power calculation with an alpha of 0.5 and a 1-beta of 0.80 to determined at least 3 mice per group should be evaluated. This explanation is provided in the methods section. Exact replicates and numbers are provided in the figure legends.

Replicates

- You should report how often each experiment was performed
- You should include a definition of biological versus technical replication
- The data obtained should be provided and sufficient information should be provided to indicate the number of independent biological and/or technical replicates
- If you encountered any outliers, you should describe how these were handled
- Criteria for exclusion/inclusion of data should be clearly stated
- High-throughput sequence data should be uploaded before submission, with a private link for reviewers provided (these are available from both GEO and ArrayExpress)

Please outline where this information can be found within the submission (e.g., sections or figure legends), or explain why this information doesn't apply to your submission:

1. The number of all biological replicates are given in the Figure legends.
2. A biologic replicate was considered a measurement of a biologically distinct sample (such as a separate mouse), a technical replicate was considered a repeated measurement of the same sample.
3. No data were excluded. All attempts at replication were successful.
4. For single cell sequencing, samples were randomly allocated into groups and unbaised clustering was performed prior to aligning samples to published data sets. Mice used were generally male or female B6 mice with no differences in gender ovserved.
This information can be found in the methods section.

Statistical reporting

- Statistical analysis methods should be described and justified
- Raw data should be presented in figures whenever informative to do so (typically when N per group is less than 10)
- For each experiment, you should identify the statistical tests used, exact values of N, definitions of center, methods of multiple test correction, and dispersion and precision measures (e.g., mean, median, SD, SEM, confidence intervals; and, for the major substantive results, a measure of effect size (e.g., Pearson's r, Cohen's d)
- Report exact p-values wherever possible alongside the summary statistics and 95% confidence intervals. These should be reported for all key questions and not only when the p-value is less than 0.05.

Please outline where this information can be found within the submission (e.g., sections or figure legends), or explain why this information doesn't apply to your submission:

1. General information on data analysis given at end of Methods section. Every figure legend has statistical information concerning the data representation, and details of tests.
2. Raw data is presented in figures
3. Error bars represent the SEM and is stated in the figure legend.
4. Exact p-values are noted in the figure legends.

(For large datasets, or papers with a very large number of statistical tests, you may upload a single table file with tests, Ns, etc., with reference to sections in the manuscript.)

Group allocation

- Indicate how samples were allocated into experimental groups (in the case of clinical studies, please specify allocation to treatment method); if randomization was used, please also state if restricted randomization was applied
- Indicate if masking was used during group allocation, data collection and/or data analysis

Please outline where this information can be found within the submission (e.g., sections or figure legends), or explain why this information doesn't apply to your submission:

For single cell sequencing, unbiased clustering was performed prior to cell type designation for antigen counting. For other analysis investigators were blinded to samples during analysis. This information can be found in the methods and figure legends.

Additional data files ("source data")

- We encourage you to upload relevant additional data files, such as numerical data that are represented as a graph in a figure, or as a summary table
- Where provided, these should be in the most useful format, and they can be uploaded as "Source data" files linked to a main figure or table
- Include model definition files including the full list of parameters used
- Include code used for data analysis (e.g., R, MatLab)
- Avoid stating that data files are "available upon request"

Please indicate the figures or tables for which source data files have been provided:

1. List of oligo sequences used can be found Figure 1-source data 1.
2. Identification cell types, number of cells, statistical test and relative antigen content within each group of cells can be found in Figure 3-source data 1. .
3. Identification of genes associated within each group of cells (eg. CCR7hi Mig cDC2) based on antigen high or antigen low cells can be found in Figure 4-source data 1. Statstics and average expression are included
4. Identification of genes associated with the larger groups of cells (eg. myeloid cells) based on antigen high or antigen low can be found in Figure 5-source data 1. Statistics and average expression are included.
5. Raw and processed data for this study have been deposited at NCBI GEO under accession GSE150719. A reproducible analysis pipeline is available at https://github.com/rnabioco/antigen-tracking.
